# Identification of diagnostic biomarkers for neonatal necrotizing enterocolitis via machine learning screening and single‐cell virtual gene knockout validation

**DOI:** 10.1002/ccs3.70081

**Published:** 2026-05-28

**Authors:** Xue Liu, Wenqiang Sun, Zengxue Hu, Yihui Li, Jingtao Bian, Xueping Zhu

**Affiliations:** ^1^ Department of Neonatology Children's Hospital of Soochow University Suzhou China; ^2^ Suzhou Medical College Soochow University Suzhou China

**Keywords:** diagnostic biomarkers, ferroptosis, immune cell infiltration, machine learning, neonatal necrotizing enterocolitis, single‐cell RNA sequencing, single‐cell virtual knockout, WGCNA

## Abstract

Neonatal necrotizing enterocolitis (NEC) continues to be the most severe gastrointestinal emergency affecting preterm infants, with reported mortality rates ranging from 20% to 30%. The absence of distinct early biomarkers results in delayed intervention and unfavorable outcomes, and the molecular mechanisms underlying the dysregulated immune response in NEC remain incompletely defined. We integrated five gene expression omnibus datasets (36 NEC cases and 34 controls) using weighted gene co‐expression network analysis and systematically evaluated 113 machine learning algorithm combinations. The best‐performing model (RF + XGBoost) identified candidate diagnostic genes, which were validated through independent bulk RNA‐sequencing cohorts, single‐cell RNA sequencing (11,308 intestinal cells), single‐cell virtual gene knockout (scTenifoldKnk), and experimental models including mice and the intestinal epithelial cells cell line. Combinatorial in silico perturbation was further performed using Geneformer, and a ferroptosis gene panel was validated by qRT‐polymerase chain reaction in the mouse NEC model. The RF + XGBoost model achieved high diagnostic accuracy (area under the curve = 0.979, 95% CI: 0.940–1.000). Three key biomarkers were identified: SLC26A3, CCL20, and CXCL5. Multi‐platform validation showed consistent downregulation of SLC26A3 and upregulation of CCL20 and CXCL5 in NEC (all *p* < 0.001). Single‐cell analyses revealed cell‐type‐specific dysregulation, with CCL20 markedly elevated in macrophages (log2FC = 3.85) and CXCL5 broadly upregulated across enterocytes, macrophages, and fibroblasts. Immune profiling demonstrated elevated proportions of M0 macrophages and activated mast cells, alongside reduced naive B cells and naive CD4 T cells, and CXCL5 expression was strongly correlated with neutrophil infiltration (*r* = 0.88, *p* < 0.001). Virtual knockout analysis revealed that perturbation of CCL20 and CXCL5 produced overlapping downstream networks converging on major histocompatibility complex class II–related antigen presentation genes, whereas SLC26A3 knockout perturbed a distinct set of epithelial barrier and innate immune genes. FTH1 was the sole gene perturbed across all three knockouts, implicating ferroptosis as a potential convergence point in NEC pathogenesis. Geneformer‐based combinatorial perturbation indicated subadditive rather than synergistic interactions among the three biomarkers, with SLC26A3 appearing as the dominant node. Extension to a 9‐gene ferroptosis panel showed coordinated directional shifts across the tested genes, and qPCR measurements in the NEC mouse ileum were consistent with the predicted expression changes for the five measured genes. SLC26A3, CCL20, and CXCL5 constitute a candidate molecular signature for early NEC diagnosis. Combinatorial perturbation analysis suggests subadditive rather than synergistic interactions among the three biomarkers, with SLC26A3 appearing as the dominant node. Virtual knockout network analyses suggest that CCL20 and CXCL5 share downstream regulatory circuits linked to antigen presentation, while SLC26A3 primarily perturbs the epithelial barrier and innate immune genes. Ferroptosis emerged as a coordinated multi‐node convergence point, with computational predictions corroborated by qPCR measurements in a mouse NEC model. These findings provide a framework for a mechanistic study and potential targeted intervention in NEC.

## Graphical abstract

Integrated machine learning screening of five gene expression omnibus datasets and 113 algorithm combinations identified three candidate diagnostic biomarkers for neonatal necrotizing enterocolitis: SLC26A3 (downregulated), CCL20, and CXCL5 (both upregulated). Single‐cell RNA sequencing localized CCL20 upregulation predominantly to macrophages. Single‐cell virtual gene knockout revealed that CCL20 and CXCL5 share downstream regulatory networks converging on major histocompatibility complex class II antigen presentation, while SLC26A3 affects distinct epithelial barrier programs—with FTH1/ferroptosis emerging as the sole convergence point across all three knockouts.

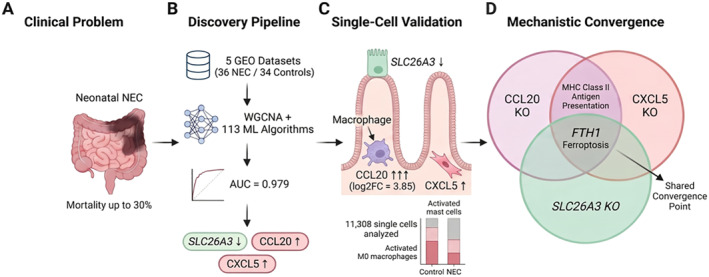

## 1 INTRODUCTION

Neonatal necrotizing enterocolitis (NEC) represents a severe acquired gastrointestinal emergency and stands as a significant cause of death among preterm infants. Present epidemiological findings suggest that NEC impacts 5%–10% of infants classified as very low birth weight, with mortality rates soaring to 30% in the most severe instances.^1^, ^2^

Even with improvements in neonatal intensive care, the occurrence of NEC appears to be on the rise, likely a result of the enhanced survival rates of extremely premature infants along with the intricate dynamics of intestinal dysbiosis.^3^ A significant clinical obstacle is that the initial stages of NEC are marked by subtle and nonspecific symptoms. Often, by the moment typical clinical indicators or radiological proof become apparent, the condition has already advanced to a stage of irreversible intestinal necrosis.

**FIGURE 1:**
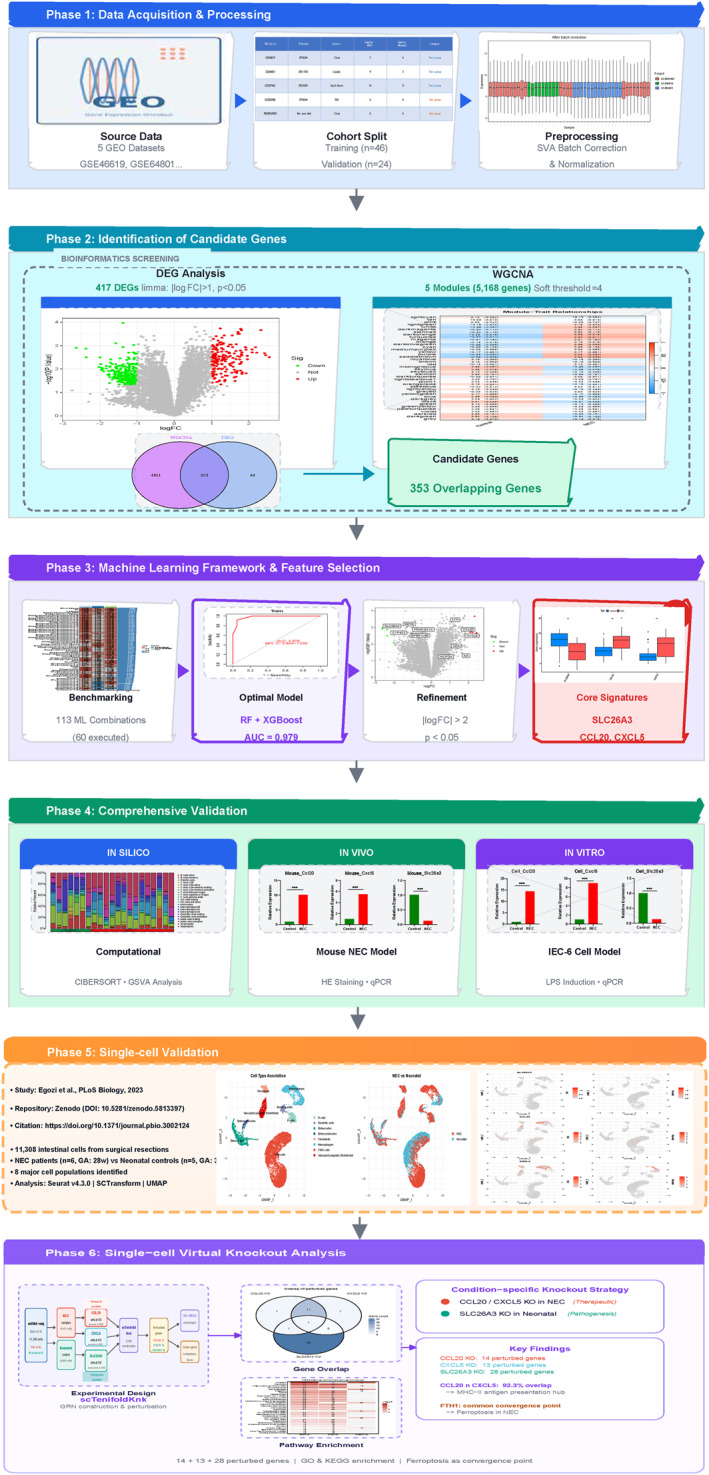
The comprehensive research workflow for identifying and validating diagnostic biomarkers in NEC. NEC, neonatal necrotizing enterocolitis.

The conventional approach to diagnosis predominantly depends on clinical evaluation and abdominal imaging. Although the modified Bell staging system is recognized as the benchmark for staging, its dependence on imaging findings from advanced stages (such as the presence of pneumatosis intestinalis) frequently leads to a “diagnostic window of missed opportunity.”^4^, ^5^ Therefore, there is a pressing necessity for consistent molecular biomarkers that can aid in early detection and risk assessment, which could potentially minimize the reliance on invasive surgical procedures. Recent findings emphasize the crucial influence of the intestinal immune microenvironment in the development of NEC. Overactive inflammatory responses, marked by abnormal infiltration of macrophages and neutrophils, along with a reduction of regulatory T cells (Tregs), result in significant mucosal injury.^6^, ^7^ Nonetheless, the specific molecular factors driving this immune imbalance are not fully clarified. Although targeted immunomodulatory treatments show potential, identifying precise molecular targets is essential for directing effective interventions. In our research, we combined multicenter gene expression omnibus (GEO) datasets and applied a comprehensive methodology that included weighted gene co‐expression network analysis (WGCNA) alongside an extensive evaluation of 113 machine learning algorithms to pinpoint essential diagnostic biomarkers (Figure 1). We additionally confirmed these biomarkers through immune infiltration assessments and laboratory models. Our aim is to establish a novel molecular framework that supports early detection and targeted therapy for NEC.

## 2 MATERIALS AND METHODS

### 2.1 Data collection and processing

All datasets related to NEC were obtained from the GEO public database (http://www.ncbi.nlm.nih.gov/geo), which includes data from projects GSE46619, GSE64801, GSE297483, GSE226086, and PRJNA925809. The criteria for selecting these datasets required that both NEC samples and normal tissue samples be included, which is essential for identifying biomarkers associated with NEC. The datasets were organized into two groups: a training group (consisting of GSE46619, GSE64801, and GSE297483, including 24 NEC cases alongside 22 normal controls) and a test group (comprising GSE226086 and PRJNA925809, containing 12 NEC cases and 12 normal controls). The test group operates as an independent validation cohort. Notably, the PRJNA925809 BioProject lacked preprocessed mRNA transcript data; however, it supplied raw sequencing data (available at https://www.ncbi.nlm.nih.gov/bioproject/PRJNA925809, with specific data processing procedures outlined in Section 2.13); thus, the BioProject number was required. Detailed information regarding the datasets employed (including methods of sample collection, sources, applications, and information about database administrators) can be found in Table 1, with all original datasets available for search in the GEO or NCBI databases.

**TABLE 1:** Basic information of GEO datasets used in this study.

GEO series	Platform	Country	Samples		Category
			NEC	Normal	
GSE46619	GPL6244	China	5	4	Train group
GSE64801	GPL11154	Canada	9	5	Train group
GSE297483	GPL20301	South Korea	10	13	Train group
GSE226086	GPL6244	USA	6	6	Test group
PRJNA925809	Not provided	China	6	6	Test group

*Note*: Five GEO datasets (GSE46619, GSE64801, GSE297483, GSE226086, and PRJNA925809) including platform, country, and sample distribution (36 NEC cases and 34 controls). Training cohort: *n* = 46; validation cohort: *n* = 24.

Abbreviations: GEO, gene expression omnibus; NEC, neonatal necrotizing enterocolitis.

### 2.2 Identification of DEGs in NEC

For normalization of raw data, the R package “limma” was employed. Data from 24 NEC cases and 22 healthy controls within the training cohort were examined, utilizing the “SVA” package for correction of batch effects. Differentially expressed genes (DEGs) were identified based on the criteria of |logFC| > 1 and *p*‐value < 0.05. To visualize all DEGs, volcano plots and clustering heat maps were produced with the R packages “ggplot2” and “pheatmap,” respectively.

### 2.3 Analysis of weighted gene Co‐expression networks

The R package “WGCNA” was employed to develop gene co‐expression networks and pinpoint modules related to NEC. Subsets of genes with *p*‐values below 0.05 were chosen for additional examination. The analysis was performed using the “goodSamplesGenes” function to eliminate missing data, discard genes or samples that fell short of quality criteria, and to build a scale‐free co‐expression network. To find the ideal soft threshold, the “pickSoftThreshold” function was applied. Consequently, the gene expression data matrix transitioned into a corresponding adjacency matrix, which was later converted into a topological overlap matrix to determine gene weights and similarities. Gene modules were identified using topological overlap clustering techniques. Module eigengenes were computed, and similar modules were merged to create hierarchical clustering dendrograms. Finally, within‐module analysis was carried out to compute gene significance (GS) and module membership (MM).

### 2.4 Functional enrichment analysis

An analysis of gene ontology (GO) and Kyoto Encyclopedia of Genes and Genomes (KEGG) was carried out on essential genes utilizing the R package “clusterProfiler,” setting a *p*‐value threshold of <0.05 as the criterion for screening. To delve deeper into the signaling pathways related to the crucial differential genes, we applied gene set variation analysis (GSVA) techniques. The GSVA was executed on the sets of differential genes using the R packages “GSVA” and “enrichplot,” referring to the gene set file “c2.cp.kegg.Hs.symbols.gmt.”

### 2.5 Diagnostic model construction using machine learning techniques

Machine learning, which falls under the broader category of artificial intelligence, primarily depends on algorithms that scrutinize data to uncover patterns and yield predictions or decisions without necessitating explicit programming.^8^ In our research, we partitioned the NEC dataset into training and testing cohorts and implemented 113 machine learning techniques. We then assessed the predictive efficiency of the models by ranking them according to their average area under the curve (AUC) values, aiming to pinpoint the most effective models and their associated genes.

### 2.6 Core biomarker identification

Tools for creating Venn diagrams were used to intersect the gene set from the optimal model with the differential gene set. The resulting intersected genes were then refined based on log fold change (logFC) and *p*‐values to determine core genes, adhering to the criteria of |logFC| > 2 and *p*‐value < 0.05. For the co‐expression network analysis of the core genes, GeneMANIA (http://www.genemania.org/) was employed.

### 2.7 Analysis of immune cell infiltration

To evaluate the composition and abundance of 22 types of immune infiltrating cells in both NEC and control samples, the CIBERSORT algorithm was utilized. The analysis of immune cell infiltration was facilitated by the R package “CIBERSORT,” and bar charts provided a clear visual representation of the proportional distribution of various immune cells across the different samples. Furthermore, the R package “ggpubr” was employed to illustrate the differences in immune cell proportions between the NEC and control groups. Additionally, correlations among the 22 infiltrating immune cell types were depicted using heat maps generated by the R package “corrplot,” with a *p*‐value threshold of <0.05 denoting statistical significance.

### 2.8 Gene identification and correlation analysis of immune cell infiltration

A Spearman rank correlation analysis was performed through R software to explore the relationships between the expression levels of identified biomarkers and the counts of infiltrating immune cells. The resulting data were visualized with the use of the “ggpubr” and “linkET” functions from R packages. A threshold for statistical significance was set at *p* < 0.05.

### 2.9 Analysis of single‐cell RNA sequencing data

To confirm the expression of target genes at a single‐cell level, we examined a publicly accessible scRNA‐seq dataset sourced from Egozi et al.,^9^ stored in the Zenodo repository (https://doi.org/10.5281/zenodo.5813397), which includes 11,308 intestinal cells obtained from patients with NEC (*n* = 6) and neonatal controls (*n* = 5). The cell type annotations and data processing were conducted using Seurat version 4.3.0.^10^ We evaluated differential expression utilizing the Wilcoxon rank‐sum test, with the Benjamini‐Hochberg method applied for false discovery rate (FDR) correction (*p* < 0.05).

### 2.10 Virtual gene knockout analysis at single‐cell level

To explore the regulatory effects of essential biomarkers on gene co‐expression networks, we executed a virtual knockout analysis employing scTenifoldKnk.^11^ This framework creates gene regulatory networks from single‐cell count matrices by means of principal component regression and simulates gene knockouts by substituting the expression of target genes with zeros, thereby pinpointing downstream genes with notably altered network embeddings.

We implemented a condition‐specific knockout strategy grounded in the expression patterns for each gene in NEC: CCL20 and CXCL5 (which are upregulated in NEC) underwent virtual knockout in NEC samples to mimic therapeutic silencing, whereas SLC26A3 (downregulated in NEC) was knocked out in samples from neonatal controls to analyze the repercussions of losing protective genes. In each analysis, the top 5000 most variable genes were chosen, and scTenifoldKnk was run with parameters set to nc_nNet = 10, nc_nCells = 500, qc_mtThreshold = 0.1, and qc_minLSize = 1000. Genes that were differentially regulated were identified using the chi‐squared test along with the Benjamini–Hochberg adjustment (adjusted *p* < 0.05). Enrichment analyses for GO and KEGG were conducted using clusterProfiler.

### 2.11 In silico perturbation with Geneformer

Geneformer^12^ was applied to the same NEC scRNA‐seq dataset for combinatorial in silico perturbation.^12^ The pretrained Geneformer‐V1‐10M model was fine‐tuned to discriminate NEC cells from neonatal control cells, with seven, two, and two patients allocated to the training, validation, and test sets respectively at the donor level (training: S1021 [NEC], S1109 [NEC], S1155 [NEC], S1193 [NEC], S1056 [Neonatal], S1127 [Neonatal], S1214 [Neonatal]; validation: S1074 [NEC], S1082 [Neonatal]; test: S1095 [NEC], S1212 [Neonatal]). Fine‐tuning was conducted for 5 epochs with a learning rate of 5 × 10^−5^ and a batch size of 4, using Python 3.10 and PyTorch 2.4.0. Single‐gene, pairwise, and triple combinatorial perturbations were performed using a custom cross‐modality framework, and effects were quantified as cosine similarity shifts of perturbed cell embeddings toward the Neonatal reference state. Synergy scores were defined as the difference between observed combinatorial shifts and the algebraic sum of the corresponding single‐gene shifts.

### 2.12 Reagents

Reagents FastPure Cell/Tissue Total RNA Extraction Kit V2, along with HiScript III RT SuperMix and ChamQ Universal SYBR qPCR Master Mix Q711‐02, were obtained from Vazyme Biotech located in Nanjing, China.

### 2.13 Experimental animals and cells

All experimental procedures in this study were approved by the Ethics Committee of Soochow University (No. SUDA20241008A01) and strictly adhered to national guidelines and regulations. C57BL/6 wild‐type mice (both male and female) were obtained from Hangzhou Ziyuan Laboratory Animal Technology Co., Ltd. At the end of the 96‐h experimental period or upon presentation of humane end points (severe abdominal distension, apnea, cyanosis, or lethargy), neonatal mice were humanely euthanized by rapid decapitation using sharp surgical scissors, in accordance with the AVMA Guidelines for the Euthanasia of Animals (2020 Edition). Terminal ileum tissues were subsequently harvested through midline laparotomy and processed for histological examination (fixed in 4% paraformaldehyde) and molecular analyses, respectively. Rat intestinal epithelial cells, recognized as a reliable non‐transformed crypt‐like cell model utilized for studying neonatal intestinal epithelial injury, were procured from Fuheng Biotechnology. This cell line was routinely maintained in a high‐glucose formulation of Dulbecco's modified Eagle's medium (DMEM) enriched with 10% fetal bovine serum (FBS) (Gibco) and 1% penicillin‐streptomycin (Gibco). Cells were kept in a 5% CO_2_ incubator set at 37°C. Upon achieving 80%–90% confluency, the cells were digested with 0.25% trypsin, passaged, and subsequently seeded into 6‐well plates according to their experimental groupings. When the cells reached approximately 80% confluency once more, they were classified into the control group and the NEC model group in alignment with the experimental goals. The culture medium for the NEC model group was substituted with a fresh medium containing 5% FBS and 30 μg/mL of lipopolysaccharide (LPS) (Sigma‐Aldrich), whereas the control group received only DMEM supplemented with 5% FBS, excluding LPS. All cells were cultured further in a 37°C incubator for an additional 24 h. Following this incubation period, either the cells or their supernatants were collected for subsequent analyses, and the successful establishment of the NEC cell model was validated by measuring the mRNA expression levels of the pro‐inflammatory cytokines IL‐6 and TNF‐α.

### 2.14 Neonatal mouse intestinal epithelial model (NEC)

Based on existing literature methods,^13^, ^14^ NEC was triggered in C57BL/6 mice on their seventh postpartum day. The grouping of animals was executed adhering to principles of randomization. The diet consisted of a mixture of Similac Advance infant formula (Abbott Nutrition) and Esbilac canine milk replacer (PetAg) in a 2:3 ratio, with 2 g of puppy milk powder combined with 3 g of infant formula dissolved in 10 mL of distilled water. This concoction was fortified with LPS at a concentration of 100 μg/mL, delivered three times daily, providing a dose of 40 μL/g. In addition, the mice underwent hypoxic stimulation twice daily for 10 min, utilizing a gas mixture comprising 95% nitrogen and 5% oxygen, followed by exposure to cold in a 4°C refrigerator for 30 min after each hypoxic session. At least 30‐min intervals separated the hypoxia from the cold exposure. The regimen of hyperosmolar formula feeding, alongside hypoxia and cold exposure, continued for a duration of 96 h. The control group was nourished with maternal milk. For hematoxylin and eosin (HE) staining, tissues from the terminal ileum of the mice were preserved in a 4% paraformaldehyde solution for a period of 24 h. After paraffin embedding, the samples were sliced to a thickness of 5 μm using a microtome. HE staining was performed to assess the extent of intestinal injury via double‐blind observation methods^15^ for scoring histological lesions. Samples that received histological scores of ≥2 were categorized as exhibiting NEC.

### 2.15 Quantitative real‐time polymerase chain reaction (PCR) analysis

Total RNA was isolated from the ileum of mice utilizing TRIzol reagent (Vazyme), with its concentration measured via a NanoDrop spectrophotometer. Following this, the RNA underwent reverse transcription to produce cDNA, employing a reverse transcription kit (HiScript III RT SuperMix for qPCR + gDNA wiper; Vazyme). The cDNA generated served as the foundational templates for the real‐time quantitative PCR, implemented with AceQ Universal SYBR qPCR Master Mix (Vazyme) on a Bio‐Rad qPCR detection system. The same protocol was further applied to a subset of three control and three NEC animals randomly selected from the cohort described above for measurement of five ferroptosis‐related genes (Gpx4, Slc7a11, Fth1, Tfrc, and Acsl4); mouse‐specific primers for these genes were appended to Supporting Information S2: Table S1. Group differences for all qPCR experiments were assessed by two‐tailed unpaired Student's *t*‐test (*p* < 0.05).

### 2.16 Statistical evaluation

All statistical evaluations were carried out using Perl version 5.38.2 alongside R software version 4.3.1. Statistical significance was determined with a *p*‐value set at a threshold of <0.05. Raw RNA sequencing data were retrieved from the NCBI SRA database (BioProject PRJNA925809) and subsequently downloaded and reformatted into a FASTQ format utilizing the SRA Toolkit (version 2.11.0). After performing quality control with FastQC (version 0.11.9), low‐quality sequences were trimmed using Trimmomatic (version 0.39) with parameters: LEADING:3, TRAILING:3, SLIDINGWINDOW:4:15, and MINLEN:36. Cleaned reads were aligned to the human reference genome GRCh38 through STAR (version 2.7.9a), while gene expression quantification was executed utilizing HTSeq (version 0.13.5).

## 3 RESULTS

### 3.1 DEGs in NEC

**FIGURE 2:**
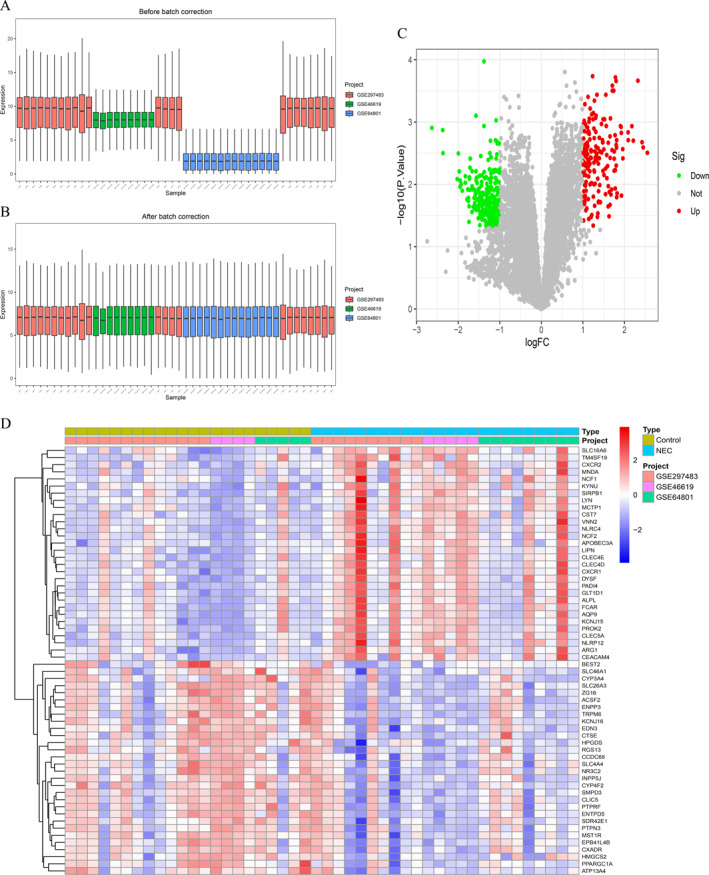
Identification of DEGs in NEC. (A and B) PCA plots showing sample distribution before (A) and after (B) batch effect correction. The SVA adjustment improved cross‐dataset uniformity. (C) Heat map of 417 DEGs (|logFC| > 1, *p* < 0.05) between 24 NEC and 22 control samples. (D) Volcano plot showing 178 upregulated (red) and 239 downregulated (blue) DEGs. Analyses were performed using the limma, ggplot2, and pheatmap R packages. DEGs, differentially expressed genes; NEC, neonatal necrotizing enterocolitis; PCA, principal component analysis; SVA, surrogate variable analysis.

Using the GEO database (accession numbers GSE46619, GSE64801, and GSE297483), we employed the R limma software package to identify DEGs in 24 NEC samples and 22 normal control samples. Due to batch effects observed between different datasets (Figure 2A), the removal of these effects resulted in a significantly improved uniformity in the distribution of the datasets (Figure 2B). This improvement indicates that, following adjustment, the integrated data can be regarded as a unified batch for subsequent analyses. Consequently, we identified a total of 417 DEGs in the training group, comprising 178 upregulated and 239 downregulated genes. The complete DEG list is provided in Supporting Information S2: Table S2. The expression levels of these genes are illustrated through heat maps and volcano plots (Figure 2C and 2D).

### 3.2 WGCNA and key module identification

**FIGURE 3:**
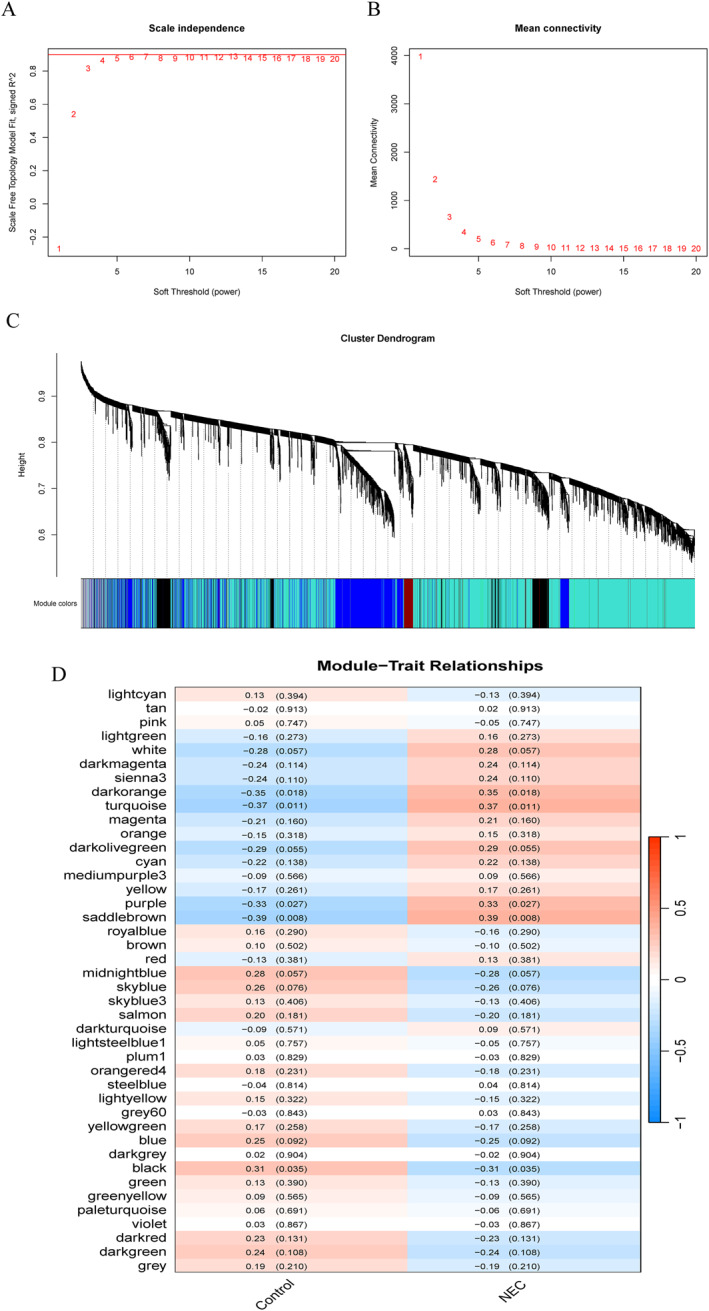
Construction of WGCNA. (A and B) Scale‐free topology and mean connectivity analysis for soft‐threshold selection. At *β* = 4, the scale‐free *R* ^2^ reached 0.9, satisfying the scale‐free network criterion. (C) Hierarchical clustering dendrogram of 42 gene modules, with distinct colors representing different modules. (D) Module–trait relationship heat map showing significant correlations between NEC and five modules (black, dark orange, purple, saddle brown, and turquoise; *R* = 0.31 to −0.39, *p* < 0.001). NEC, neonatal necrotizing enterocolitis; WGCNA, weighted gene co‐expression network.

**FIGURE 4:**
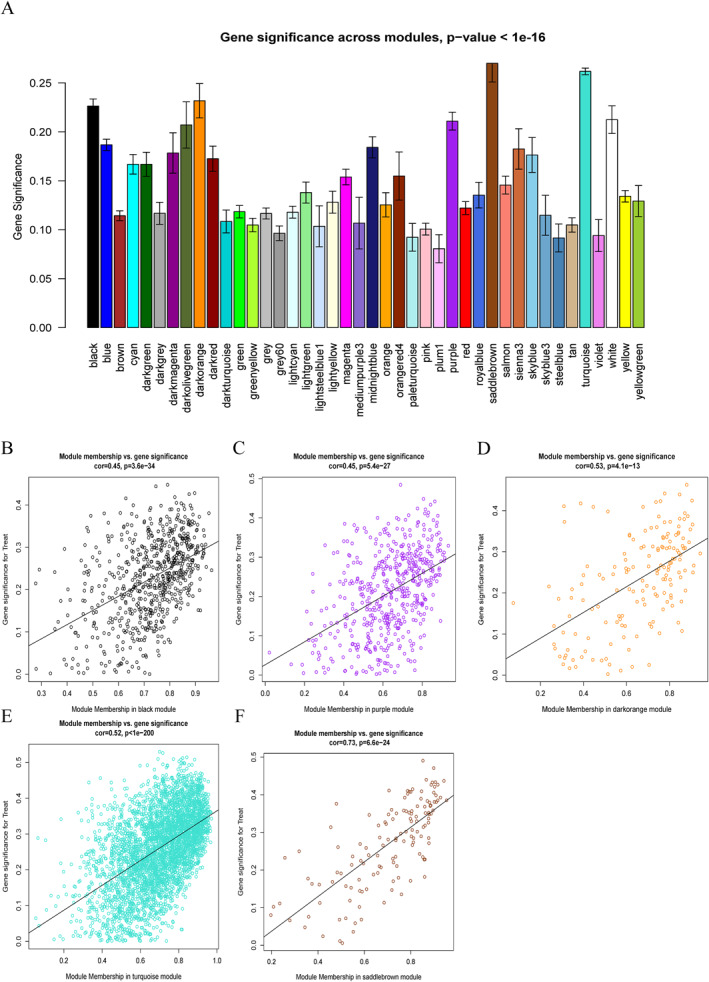
Module significance and gene significance analysis. (A) Distribution of GS across 42 modules. (B–F) Scatterplots showing strong positive correlations between MM and GS in five key modules (black, dark orange, purple, saddle brown, and turquoise; *R* > 0.3, *p* < 0.001), indicating their importance in NEC. GS, gene significance; MM, module membership; NEC, neonatal necrotizing enterocolitis.

We employed WGCNA methods to construct scale‐free co‐expression networks for identifying modules most strongly associated with neonatal NEC. Based on the criteria of scale independence and mean connectivity indices, we selected an optimal soft threshold of *β* = 4 (scale‐free *R* ^2^ = 0.9) (Figure 3A and 3B). Utilizing dynamic tree cutting, we identified 42 distinct colored modules (Figure 3C). Subsequently, by analyzing *p*‐values and correlation coefficients of these modules, we determined that five colored modules (black, purple, dark orange, turquoise, and saddle brown) exhibited significant associations with NEC (*R* = −0.31, *R* = 0.33, *R* = 0.35, *R* = 0.37, and *R* = 0.39, respectively; all *p* < 0.05) (Figure 3D). A significant distribution of GS was observed across the 42 modules (Figure 4A). Consequently, we selected 5168 genes from these five modules for further analysis (Supporting Information S2: Table S3). Additionally, we conducted a correlation analysis between MM and GS, revealing significant positive correlations between these genes and their respective modules (Figure 4B–F).

### 3.3 Functional enrichment analysis

**FIGURE 5:**
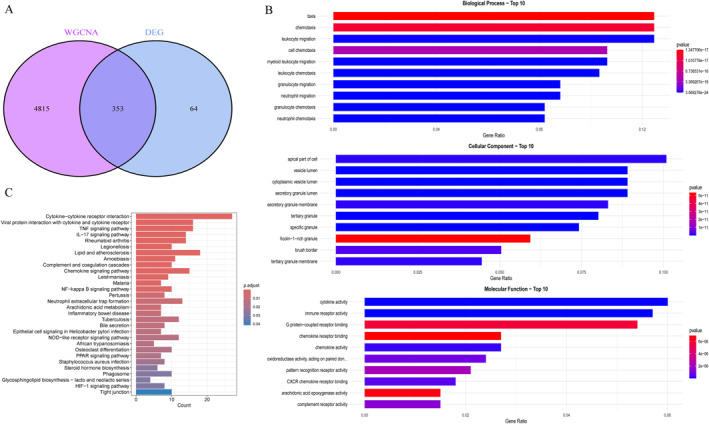
Functional enrichment analysis of core genes. (A) Venn diagram showing the intersection of DEGs and WGCNA‐derived genes, identifying 353 candidate genes. (B) GO enrichment results: BP were enriched in neutrophil migration and chemotaxis; CCs included secretory and specific granules; MFs involved cytokine and chemokine binding. (C) KEGG pathway analysis revealed enrichment in cytokine–cytokine receptor interaction and IL‐17 signaling pathways (*p* < 0.05). BP, biological processes; CCs, cellular components; DEGs, differentially expressed genes; GO, gene ontology; KEGG, Kyoto Encyclopedia of Genes and Genomes; MFs, molecular functions; WGCNA, weighted gene co‐expression network.

We conducted a cross‐comparison of DEGs with WGCNA module genes, identifying a total of 353 genes (Figure 5A). The expression patterns of these genes are presented in Supporting Information S2: Table S4. To further assess the potential regulatory pathways associated with these core differential genes in NEC, we performed GO and KEGG enrichment analyses. The results of the GO analysis are summarized in Supporting Information S2: Table S5, indicating that these genes are predominantly enriched in biological processes (BP) such as “neutrophil migration,” “neutrophil chemotaxis,” and “leukocyte migration.” In the cellular component ontology, they were also linked to “secretory granules” and “specific granules.” The molecular function (MF) analysis revealed that these genes were enriched in terms such as “cytokine binding” and “chemokine binding” (Figure 5B). The findings of the KEGG enrichment analysis are displayed in Supporting Information S2: Table S6, demonstrating that these genes primarily participate in pathways such as “cytokine‐cytokine receptor interaction” and the “IL‐17 signaling pathway” (Figure 5C).

### 3.4 Identification of candidate core genes using machine learning methods

**FIGURE 6:**
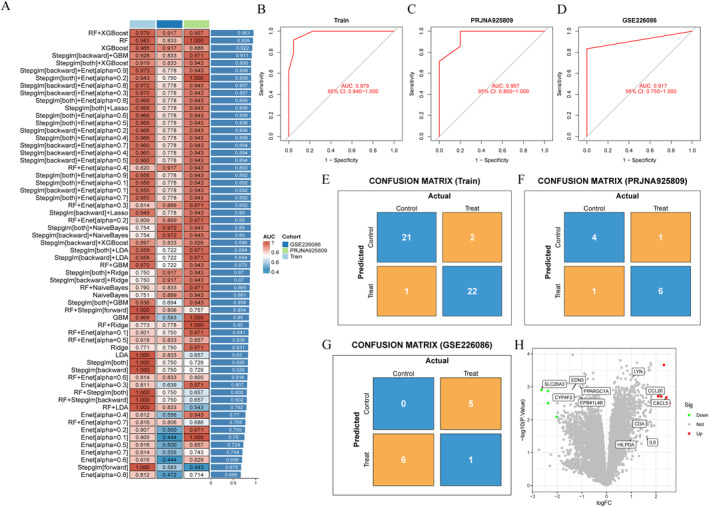
Machine learning model construction and core gene selection. (A) Comparison of AUC values for 113 machine learning algorithms in the training set, showing the RF + XGBoost model achieved the best performance (AUC = 0.979, 95% CI: 0.940–1.000). (B–D) ROC curves for RF + XGBoost in the training set, GSE226086, and PRJNA925809 test cohorts, with AUCs of 0.979, 0.917, and 0.957, respectively. (E–G) Confusion matrices showing prediction accuracy >90% across cohorts. (H) Volcano plot highlighting three final core genes (SLC26A3, CCL20, and CXCL5; |logFC| > 2, *p* < 0.05). AUC, area under the curve; ROC, receiver operating characteristic.

To construct a diagnostic model for NEC‐related genes, we employed 113 machine learning algorithms to analyze a pre‐screened set of 353 genes, of which 60 algorithms successfully executed. In the training dataset, the “RF + XGBoost” algorithm exhibited the best predictive performance among all tested algorithms, and this model also demonstrated excellent performance in the test dataset. The gene set associated with the model is detailed in Supporting Information S2: Table S7. Additionally, we calculated the AUC values for various models in both the training and validation groups, with results indicating that RF + XGBoost achieved the highest average AUC value (Figure 6A). Consequently, we identified the RF + XGBoost model as the optimal diagnostic model. Furthermore, we generated receiver operating characteristic (ROC) curves and confusion matrix plots for the RF + XGBoost model in the training set and two independent validation sets (GSE226086 and PRJNA925809) (Figure 6B–G). This model selected 92 gene variables. To further refine the variable selection, we applied the criteria of |logFC| > 2 and *p*‐value < 0.05 to the 92 variables, ultimately identifying three validated core genes (SLC26A3, CCL20, and CXCL5) (Figure 6H).

### 3.5 Diagnostic evaluation and validation of core genes

**FIGURE 7:**
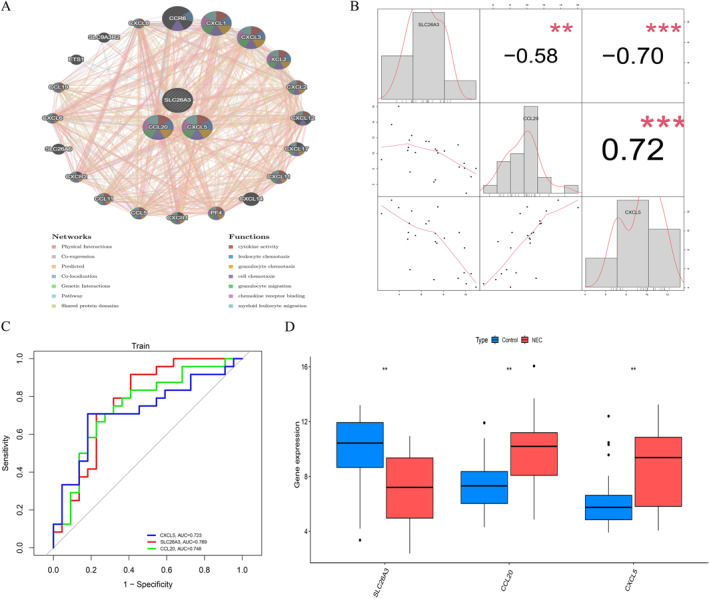
Diagnostic value and expression of core genes. (A) GeneMANIA network showing interactions between the three core genes and 20 related genes. (B) Correlation analysis: SLC26A3 negatively correlated with CXCL5 (*r* = −0.70), while CCL20 positively correlated with CXCL5 (*r* = 0.65). (C) ROC curves for diagnostic performance of SLC26A3 (AUC = 0.769), CCL20 (AUC = 0.748), and CXCL5 (AUC = 0.723). (D) Boxplots showing upregulation of CCL20 and CXCL5 and downregulation of SLC26A3 in NEC samples (**p < 0.01). AUC, area under the curve; NEC, neonatal necrotizing enterocolitis; ROC, receiver operating characteristic.

To thoroughly investigate the biological significance of these three core genes, we utilized GeneMANIA software to analyze the associations and interaction patterns among these genes and other closely related genes, identifying 20 genes that are closely associated with them. Functional enrichment analysis indicated that these genes predominantly participate in BP such as leukocyte chemotaxis, granulocyte chemotaxis, and cell chemotaxis, with significant enrichment observed in MFs, including cytokine activity and chemokine receptor binding. Additionally, these genes were closely linked to immune‐associated pathways, such as granulocyte migration and myeloid leukocyte migration, suggesting that they may play crucial roles in the pathogenesis of NEC through the regulation of immune cell recruitment and migration (Figure 7A). Correlation analysis demonstrated that SLC26A3 exhibited significant negative correlations with CXCL5 and CCL20 expression (*r* = −0.70 and *r* = −0.58, respectively), while a significant positive correlation was found between CCL20 and CXCL5 (Figure 7B). To assess the diagnostic value of these three candidate core genes, we established ROC curves to evaluate their diagnostic efficacy in the training group. The areas under the ROC curves for each gene were as follows: SLC26A3 (AUC = 0.769), CCL20 (AUC = 0.748), and CXCL5 (AUC = 0.723), with all candidate genes demonstrating moderate to good diagnostic value for NEC (Figure 7C). Furthermore, within the training set, we compared the expression differences of the three genes between the experimental and control groups, revealing significant upregulation of CCL20 and CXCL5, alongside significant downregulation of SLC26A3 (Figure 7D). The areas under the ROC curves for the three indicators in the validation set ranged from 0.829 to 1.000 (Supporting Information S1: Figure S1A and B). Using GSVA methods, we conducted KEGG database pathway enrichment analysis for each gene, demonstrating that CXCL5, SLC26A3, and CCL20 were primarily enriched in immune inflammation‐related pathways, including the toll‐like receptor signaling pathway, cytokine–cytokine receptor interaction, and NOD‐like receptor signaling pathway (Supporting Information S1: Figure S2A–C).

### 3.6 Immune cell infiltration analysis

**FIGURE 8:**
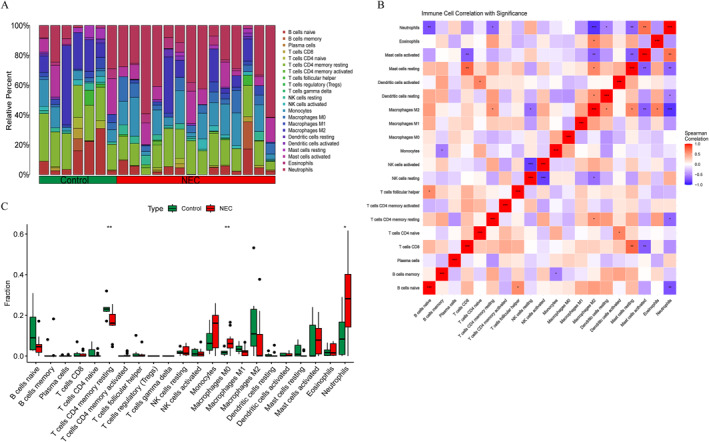
Immune cell infiltration analysis. (A) Stacked bar chart showing proportions of 22 immune cell subsets in the training cohort. (B) Correlation heat map showing a strong negative correlation between M2 macrophages and neutrophils (*r* = −0.875 and *p* < 0.001). (C) Boxplots showing significantly higher proportions of M0 macrophages and activated mast cells, and reduced proportions of naïve B cells and naïve CD4 T cells in NEC compared with controls (*p < 0.05, **p < 0.01). NEC, neonatal necrotizing enterocolitis.

We employed the CIBERSORT algorithm to evaluate the composition and abundance of 22 types of immune cell infiltration within the training group (Figure 8A). Correlation analysis revealed a highly significant negative correlation between M2 macrophages and neutrophils (*r* = −0.875 and *p* < 0.001). Additionally, naive B cells showed a significant negative correlation with neutrophils (*r* = −0.753 and *p* = 0.002), while naive CD4 T cells exhibited a significant positive correlation with activated dendritic cells (*r* = 0.627 and *p* = 0.016) (Figure 8B). Specific correlation data are detailed in Supporting Information S2: Table S8. Box plots indicated that compared to the control group, the NEC group displayed significantly elevated proportions of M0 macrophages and activated mast cells, while the proportions of naive B cells and naive CD4 T cells were significantly reduced (Figure 8C).

### 3.7 Correlation analysis between candidate biomarker gene expression and immune infiltrating cells

**FIGURE 9:**
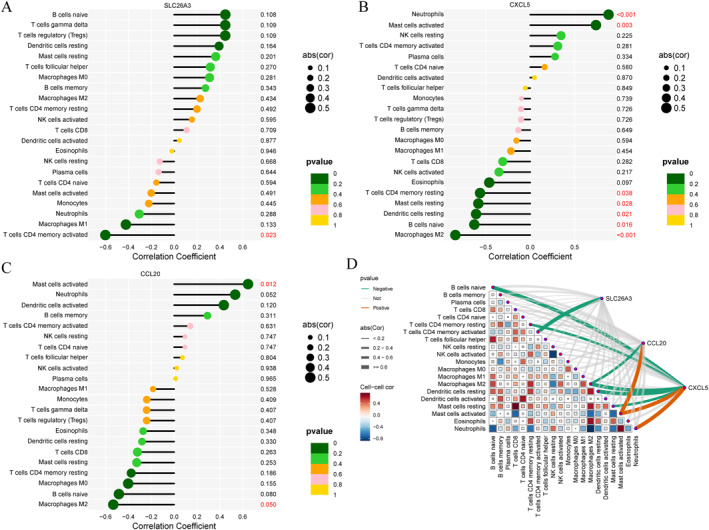
Correlations between core genes and immune cell infiltration. (A) SLC26A3 negatively correlated with activated memory CD4 T cells (*R* = −0.60 and *p* = 0.023). (B) CCL20 positively correlated with activated mast cells (*R* = 0.65 and *p* = 0.012) and negatively correlated with M2 macrophages (*R* = −0.53 and *p* = 0.050). (C) CXCL5 positively correlated with neutrophils (*R* = 0.88 and *p* < 0.001) and negatively correlated with resting mast cells (*R* = −0.58, *p* = 0.028). (D) Heat map summarizing correlations between the three genes and 22 immune cell subsets.

To validate the relationship between core genes and immune cell infiltration, we conducted a correlation analysis. The results indicated that SLC26A3 exhibited a significant negative correlation with activated CD4 memory T cells (*R* = −0.60 and *p* = 0.023) (Figure 9A). Conversely, CCL20 demonstrated a significant positive correlation with activated mast cells (*R* = 0.65 and *p* = 0.012) and a negative correlation with M2 macrophages (Figure 9B). Furthermore, CXCL5 displayed a highly significant positive correlation with neutrophils (*R* = 0.88 and *p* < 0.001) and significant negative correlations with M2 macrophages (*R* = −0.84 and *p* < 0.001) and resting mast cells (*R* = −0.58 and *p* = 0.028) (Figure 9C). Additionally, we illustrated the correlations between the expression levels of all core genes and the infiltration degrees of 22 immune cell types through visualization analysis (Figure 9D). These findings suggest that core genes may play a role in disease progression by regulating specific immune cell infiltration.

### 3.8 Validation of candidate biomarker genes in cell and animal models

**FIGURE 10:**
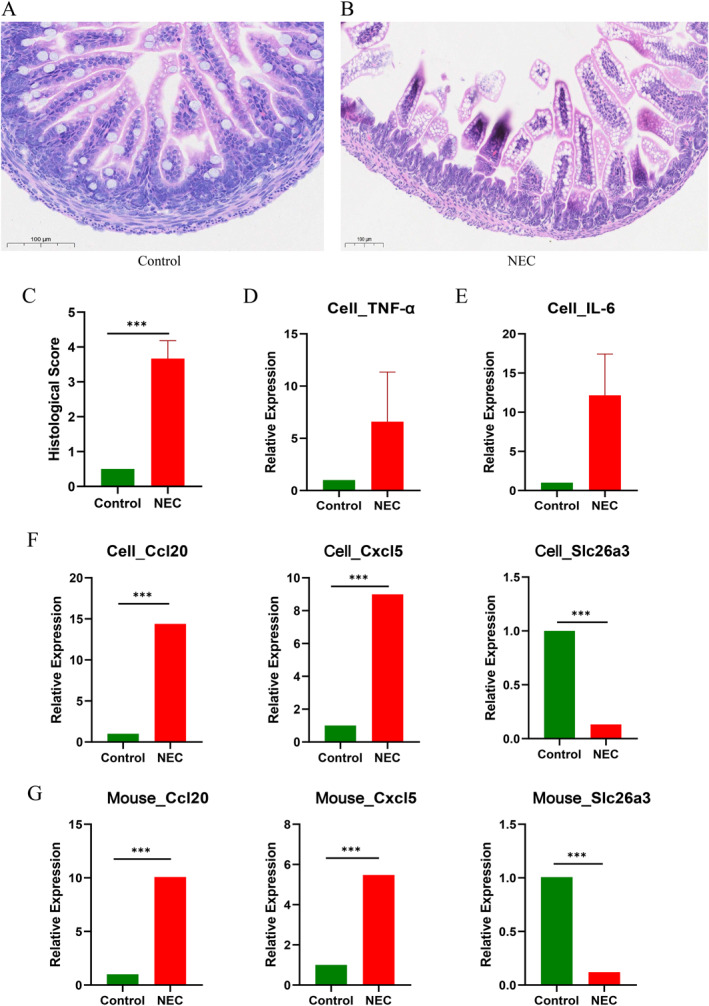
Validation in mouse and cell models. (A–C) HE staining of ileal tissue from NEC mice showing villus atrophy, epithelial shedding, and thinning of the muscular layer, with significantly higher histological scores than controls (***p < 0.001). (D and E) NEC cell models showed significantly elevated IL‐6 and TNF‐α mRNA expression. (F and G) qPCR validation demonstrated downregulation of SLC26A3 and upregulation of CCL20 and CXCL5 in NEC tissues and cells (***p < 0.001). Data are presented as mean ± SEM, and *n* = 6 per group, analyzed by *t*‐test. HE, hematoxylin and eosin; NEC, neonatal necrotizing enterocolitis; SEM, standard error of the mean.

We constructed NEC models using IEC6 cells and mice, respectively. The pathological sections of the animal model are illustrated in the figures, which clearly demonstrate that the NEC group (*n* = 6) exhibited significantly reduced villi, increased epithelial cell shedding, and thinned muscular layers compared to the control group (*n* = 6). Additionally, the intestinal pathology scores were higher in the NEC group than in the control group, indicating the successful establishment of the NEC mouse model (Figure 10A–C). In the NEC cell model, the expression levels of IL‐6 and TNF‐α were significantly elevated, further confirming the successful establishment of the cell model (Figure 10D and 10E). The detection of mRNA expression levels of screened genes in both cells and animal tissues revealed that compared to normal tissues or cells, SLC26A3 expression levels were significantly downregulated in diseased tissues or cells, while CCL20 and CXCL5 were significantly upregulated. These findings suggest that SLC26A3, CCL20, and CXCL5 play crucial roles in disease progression (Figure 10F,G; Supporting Information S2: Tables S13 and S14).

### 3.9 Single‐cell RNA‐seq validation of target biomarkers

**FIGURE 11:**
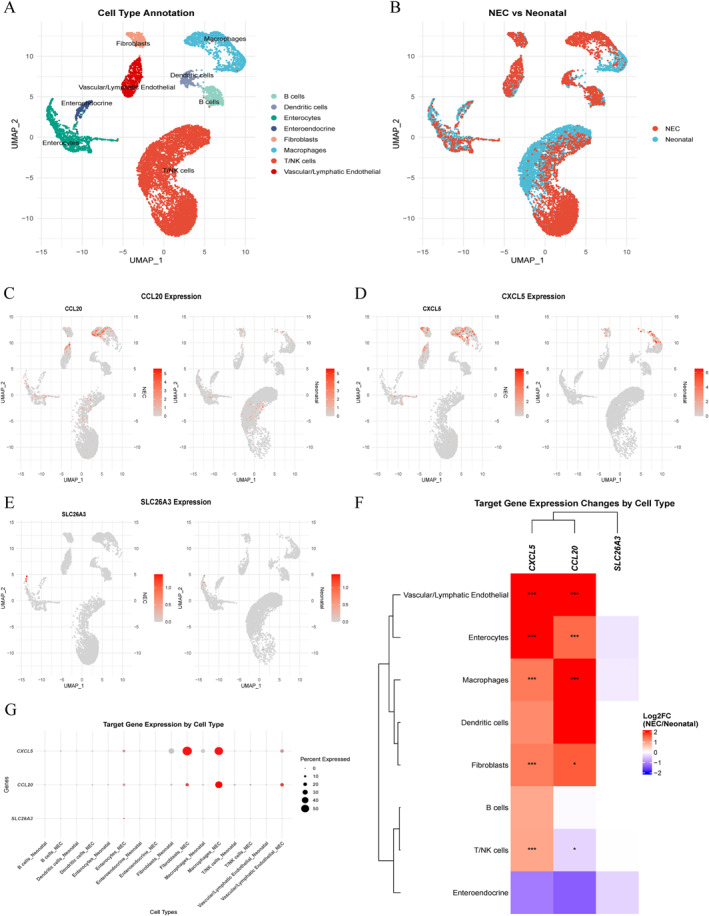
Single‐cell validation of target genes in NEC. (A, B) UMAP visualization of 11,308 intestinal cells showing cell type annotation (A) and condition distribution (B) Red = NEC, and blue = neonatal. Eight major cell populations were identified. (C–E) Expression patterns of CCL20 (C), CXCL5 (D), and SLC26A3 (E) in NEC versus neonatal samples. Color intensity represents normalized expression level (gray = low and red = high). (F) Heat map of log2 fold changes (NEC/neonatal) across cell types. *p < 0.05 and ***p < 0.001 (Wilcoxon test and FDR correction). (G) Cell type‐specific expression. Dot size indicates percentage of expressing cells; color shows average expression level. All three genes showed significant differential expression (FDR < 0.001). FDR, false discovery rate; NEC, necrotizing enterocolitis; UMAP, Uniform Manifold Approximation and Projection.

To validate our findings at single‐cell resolution, we analyzed a publicly available scRNA‐seq dataset comprising 11,308 intestinal cells from NEC and neonatal control tissues (Figure 11A,B). Consistent with bulk RNA‐seq and experimental validation, single‐cell analysis confirmed significant differential expression of all three target genes: SLC26A3 was downregulated (log2FC = −0.30 and FDR < 0.001), while CCL20 and CXCL5 were markedly upregulated (log2FC = 1.86 and 2.24 and FDR < 10^−40^) (Figure 11C–E, Supporting Information S2: Table S9). Cell type‐specific analysis revealed that CCL20 showed highest upregulation in macrophages (log2FC = 3.85 and FDR < 10^−54^), while CXCL5 exhibited widespread upregulation across enterocytes, macrophages, and fibroblasts (all FDR < 10^−7^) (Figure 11F and 11G), supporting their roles in coordinating epithelial–immune interactions during NEC pathogenesis.

### 3.10 Virtual knockout reveals distinct regulatory networks for core biomarkers

**FIGURE 12:**
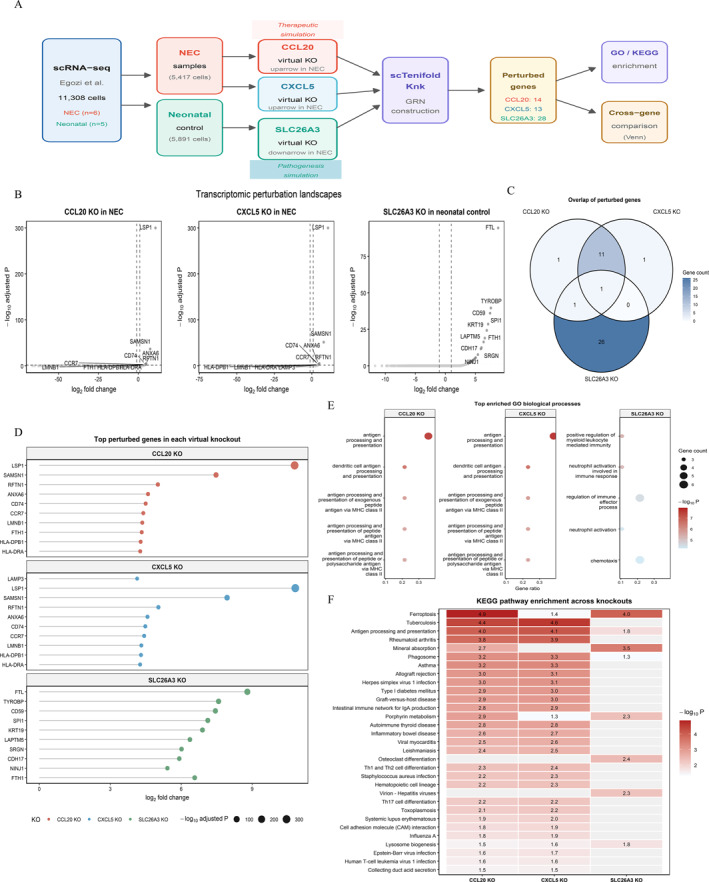
Single‐cell virtual gene knockout analysis of NEC core biomarkers. (A) Schematic of the condition‐specific virtual knockout design. CCL20 and CXCL5 (upregulated in NEC) were knocked out in NEC samples to simulate therapeutic silencing; SLC26A3 (downregulated in NEC) was knocked out in neonatal control samples to model pathogenic consequences of protective gene loss. (B) Volcano plots displaying differentially regulated genes for CCL20 knockout (left), CXCL5 knockout (middle), and SLC26A3 knockout (right). (C) Venn diagram showing overlap of significantly perturbed genes across three virtual knockouts. CCL20 and CXCL5 shared 12 genes, with FTH1 as the sole gene common to all three. (D) Top perturbed genes ranked by log_2_ fold change for each knockout. Point color and size indicate –log_10_ (adjusted *p*‐value). (E) Comparative bubble plot of top enriched GO biological process terms. CCL20 and CXCL5 knockouts converge on antigen processing and presentation, while SLC26A3 knockout enriches neutrophil activation and chemotaxis. (F) Cross‐knockout KEGG pathway enrichment heat map. Values represent –log_10_ (*p*‐value). Ferroptosis was significantly enriched in both CCL20 and SLC26A3 knockouts. Gray cells indicate nonsignificant enrichment. GO, gene ontology; KEGG, Kyoto Encyclopedia of Genes and Genomes; NEC, necrotizing enterocolitis.

To explore the functional consequences of core gene perturbation at the network level, we performed condition‐specific virtual knockout analysis (Figure 12A).

Virtual knockout of CCL20 in NEC samples identified 14 significantly perturbed genes (adjusted *p* < 0.05), with LSP1 (FC = 1774.5) and SAMSN1 (FC = 177.4) showing the strongest perturbation (Figure 12B–D, Supporting Information S1: Figure S3A, and Supporting Information S2: Table S10). The perturbed gene set was enriched in major histocompatibility complex (MHC) class II components (HLA‐DPB1, HLA‐DRA, and CD74) and CCR7. GO analysis revealed predominant involvement in antigen processing and presentation (*p* = 1.83 × 10^−8^) and T cell activation (*p* = 3.52 × 10^−5^). KEGG analysis identified enrichment in ferroptosis (*p* = 1.35 × 10^−5^) and antigen processing and presentation (*p* = 1.01 × 10^−4^) (Figure 12E and 12F, Supporting Information S1: Figure S3B, and Supporting Information S2: Tables S11 and S12). Notably, SLC7A11, a key ferroptosis inhibitor, was uniquely perturbed.

CXCL5 knockout in NEC samples identified 13 perturbed genes, of which 12 (92.3%) overlapped with CCL20 results (Figure 12B and Supporting Information S1: Figure S3C and D). The single unique gene was REL, a NF‐κB subunit (FC = 13.7 and adjusted *p* = 0.047), with enrichment in NF‐κB signal transduction (*p* = 4.80 × 10^−4^).

In contrast, SLC26A3 knockout in neonatal control tissue identified 28 perturbed genes with a distinct profile (Figure 12B–D and Supporting Information S1: Figure S3E,F), comprising epithelial barrier genes (KRT19, EPCAM, CLDN4, and CDH17) and innate immune activators (TYROBP, SPI1, S100A9, and FCER1G). GO analysis highlighted neutrophil activation (*p* = 5.09 × 10^−6^) and cell–cell junction assembly (*p* = 1.74 × 10^−3^). KEGG analysis identified ferroptosis (*p* = 1.05 × 10^−4^) and mineral absorption (*p* = 3.21 × 10^−4^) (Figure 12E,F).

Cross‐comparison revealed that CCL20 and CXCL5 converge on a shared MHC class II–centered network, while SLC26A3 perturbation primarily affected epithelial integrity and innate immunity genes (Figure 12C). FTH1 was the only gene perturbed across all three knockouts, implicating ferroptosis as a common convergence point.

### 3.11 Geneformer reveals SLC26A3 dominance and subadditive interactions

**FIGURE 13:**
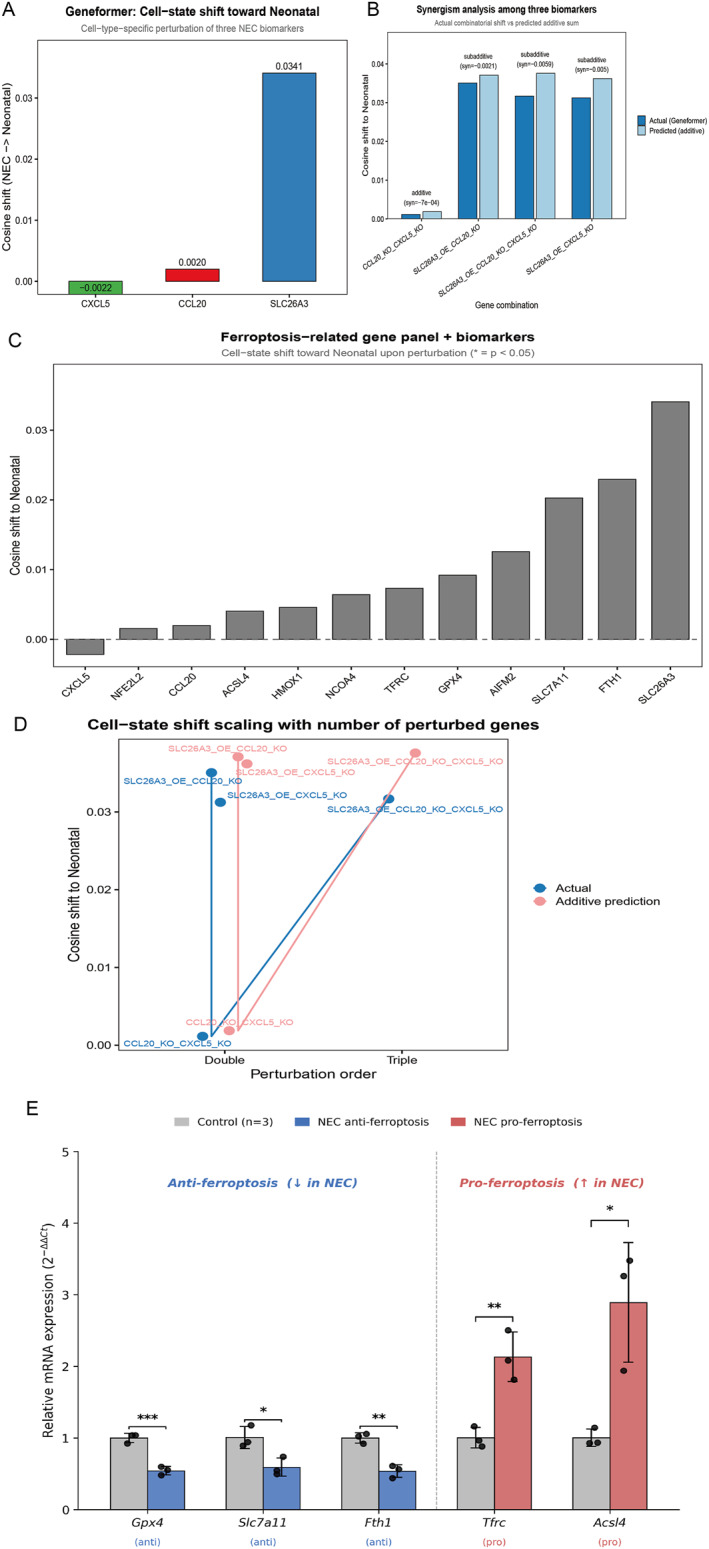
Geneformer‐based in silico perturbation reveals SLC26A3 dominance, subadditive network interactions, and a multigene ferroptosis convergence axis validated by qPCR in a mouse NEC model. (A) Cell state shifts toward the Neonatal embedding upon cell‐type‐specific single‐gene perturbation of the three NEC biomarkers (SLC26A3 overexpression in enterocytes, CCL20 deletion in macrophages, and CXCL5 deletion in enterocytes, macrophages, and fibroblasts). (B) Synergism analysis comparing actual cell state shifts (dark blue) versus additive predictions from single‐gene effects (light blue). Synergy scores below zero indicate subadditive interactions. (C) Expanded perturbation panel of 9 ferroptosis‐related genes alongside the 3 biomarkers. Anti‐ferroptosis genes (orange) were overexpressed; pro‐ferroptosis genes (brown) were deleted. (D) Cell state shift scaling with combinatorial complexity (double vs. triple combinations), showing a widening gap between actual and additive predictions. (E) qPCR validation of five ferroptosis regulators in the mouse NEC ileum (*n* = 3 per group, sampled from the Figure 10F biomarker cohort). Anti‐ferroptosis genes (Gpx4, Slc7a11, and Fth1) were significantly downregulated; pro‐ferroptosis genes (Tfrc and Acsl4) were significantly upregulated. Mean ± SEM; *p < 0.05, **p < 0.01, and ***p < 0.001 (unpaired *t*‐test). NEC, necrotizing enterocolitis; SEM, standard error of the mean.

To distinguish whether the three biomarkers act synergistically, additively, or redundantly, we performed combinatorial in silico perturbation with Geneformer and compared the observed combinatorial effects against the additive sum of single‐gene effects. Single‐gene perturbation revealed marked heterogeneity in rescue capacity. SLC26A3 overexpression in NEC enterocytes produced the strongest shift toward the Neonatal embedding, with a cosine shift of 0.034, whereas CCL20 deletion in macrophages and CXCL5 deletion produced only minimal effects, with cosine shifts of 0.002 and −0.002, respectively (Figure 13A). All combinatorial perturbations were subadditive rather than synergistic. The triple combination produced a shift of 0.032, lower than the value of 0.038 expected from additive single‐gene effects, corresponding to a synergy score of −0.006 (Figure 13B–D). These results indicate that the three biomarkers occupy partially redundant positions within a common regulatory pathway, with SLC26A3 appearing as the dominant node.

### 3.12 Convergent ferroptosis axis beyond FTH1

Our original scTenifoldKnk analysis identified FTH1 as the sole ferroptosis‐related convergence node, raising the question of whether ferroptosis suppression in NEC operates through this single iron storage hub or through a broader pathway‐level program. To distinguish between these possibilities, we tested the directional prediction that if a coordinated ferroptosis‐suppression axis is engaged, opposing perturbations of mechanistically distinct ferroptosis genes should drive cells in the same direction toward the Neonatal state. We therefore applied Geneformer to a 9‐gene ferroptosis panel in NEC enterocytes, macrophages, and fibroblasts, overexpressing six anti‐ferroptosis genes and deleting three pro‐ferroptosis genes (Figure 13C and Supporting Information S2: Table S15). All nine perturbations produced positive cosine shifts toward the Neonatal state, with the three strongest hits being FTH1 (0.023), SLC7A11 (0.020), and AIFM2 (0.013). These genes span mechanistically distinct ferroptosis suppression pathways including iron storage, cystine import, and CoQ10‐mediated radical scavenging, suggesting that ferroptosis suppression in NEC may operate as a coordinated multi‐node program rather than through FTH1 alone.

### 3.13 qPCR validation of the ferroptosis axis in mouse NEC

To experimentally confirm the predicted ferroptosis axis dysregulation, we quantified mRNA expression of the five top‐ranked ferroptosis genes by qRT‐PCR in the mouse ileum (*n* = 3 per group, sampled from the Figure 10F biomarker cohort). All five genes showed the directional changes predicted by Geneformer (Figure 13E; Supporting Information S2: Table S16). The anti‐ferroptosis genes were significantly downregulated in NEC (Gpx4 fold change 0.54, *p* = 0.0008; Slc7a11 0.59, *p* = 0.023; and Fth1 0.54, *p* = 0.002), and the pro‐ferroptosis genes were significantly upregulated (Tfrc 2.12, *p* = 0.007 and Acsl4 2.88, *p* = 0.018), supporting the ferroptosis suppression axis identified in silico.

## 4 DISCUSSION

NEC still lacks reliable early diagnostic markers, and clinical diagnosis often relies on imaging findings that appear only after the disease has progressed to irreversible intestinal necrosis. In this study, we integrated multicenter GEO transcriptomic data and applied WGCNA combined with 113 machine learning algorithm combinations, ultimately identifying SLC26A3, CCL20, and CXCL5 as core diagnostic candidate genes for NEC. All three genes were consistently validated across independent cohorts, single‐cell RNA‐seq data, and experimental models, supporting their potential as stable molecular markers for early NEC diagnosis.

SLC26A3 is an intestinal epithelial cell‐specific chloride/bicarbonate exchanger essential for intestinal electrolyte homeostasis. In the present study, SLC26A3 was significantly downregulated in NEC tissues and cell models, with single‐cell analysis confirming this trend and showing a negative correlation with activated CD4 memory T cells. Kumar et al.^16^ reported that SLC26A3 deficiency reduced tight junction protein expression (ZO‐1 and occludin) through CUGBP1‐mediated post‐transcriptional regulation, increasing intestinal permeability. Kini et al.^17^ similarly observed microbiota dysbiosis and elevated TNF‐α in SLC26A3 knockout mice. Our virtual knockout experiment revealed perturbation of epithelial barrier genes (KRT19, EPCAM, CLDN4, and CDH17) and myeloid immune activators (TYROBP, SPI1, and S100A9), consistent with the epithelial‐immune cross talk reported by Jayawardena et al.^18^ These findings suggest that SLC26A3 downregulation extends beyond an epithelial event. Once the barrier is compromised, luminal bacteria and their products penetrate the epithelium, triggering innate immune overactivation and initiating a “barrier damage‐immune activation” cascade. The concurrent enrichment of the ferroptosis pathway in this knockout analysis hints at an underappreciated connection between barrier dysfunction and ferroptosis.^19^, ^20^ From a translational standpoint, SLC26A3 downregulation may serve as an early signal of barrier impairment, and restoring its expression through microbiota modulation or epigenetic approaches deserves future exploration.^17^, ^21^

CCL20 functions as a chemotactic regulatory hub in intestinal inflammation. Our results showed significant CCL20 upregulation in NEC, with positive correlation to activated mast cells and negative correlation to M2 macrophages. Single‐cell analysis revealed the most pronounced upregulation in macrophages, suggesting them as a major cellular source. While CCL20 classically recruits Th17 cells via CCR6,^22^ its close association with mast cell activation in our study points to a less conventional regulatory pathway in NEC. Activated mast cells release histamine and tryptase that increase vascular permeability and compromise microvascular integrity.^23^ At the transcriptional level, NF‐κB binding sites in the CCL20 promoter allow inflammatory factors such as TNF‐α and LPS to upregulate its expression,^24^ creating a positive feedback loop where CCL20‐driven mast cell activation releases mediators that further reactivate NF‐κB. Virtual knockout of CCL20 perturbed MHC class II molecules (HLA‐DPB1, HLA‐DRA, and CD74) and CCR7, with enrichment in antigen processing and T cell activation, suggesting that CCL20 may participate in antigen presentation regulation beyond its chemotactic function. The specific perturbation of ferroptosis inhibitor SLC7A11, together with recent reports linking ferroptosis to NEC epithelial injury^19^ and macrophage inflammation,^20^ suggests that CCL20 downstream networks may extend into ferroptosis regulation. Stallhofer et al.^22^ have reported that vitamin D metabolites suppress CCL20 expression, offering a potential direction for therapeutic strategies targeting the CCL20‐CCR6 axis combined with ferroptosis inhibition.

CXCL5 mediates neutrophil directional migration through CXCR2 receptor binding.^25^, ^26^ In our study, CXCL5 was significantly upregulated in NEC with strong positive correlation to neutrophil infiltration and negative correlation to M2 macrophages. Single‐cell data showed widespread CXCL5 upregulation across enterocytes, macrophages, and fibroblasts, suggesting a broad‐spectrum signaling role in epithelial–immune interactions. Feng et al.^27^ demonstrated that CXCR2 antagonists effectively blocked excessive neutrophil infiltration and reduced intestinal damage in neonatal NEC mice, directly supporting the pathogenic involvement of this axis. The negative correlation with M2 macrophages suggests that CXCL5 may sustain the inflammatory microenvironment through a dual mechanism, promoting pro‐inflammatory neutrophil infiltration while suppressing anti‐inflammatory M2 macrophage recruitment.^28^ Virtual knockout analysis showed extensive overlap between CXCL5 and CCL20 downstream perturbation profiles, converging on the MHC class II antigen presentation network, while the NF‐κB subunit REL was uniquely perturbed by a CXCL5 knockout. This suggests functional convergence of these two chemokines at the adaptive immune level despite their different receptor specificities,^29^ with CXCL5 potentially regulating a distinct downstream network through NF‐κB signaling. Given its strong association with neutrophils, CXCL5 may serve as an indicator of inflammatory activity in NEC, and CXCL5‐CXCR2 targeted strategies warrant further clinical investigation.^27^

Immune infiltration analysis uncovered an imbalanced landscape in NEC intestinal tissues, with elevated M0 macrophages and activated mast cells alongside reduced naive B cells and naive CD4 T cells, reflecting “innate immune hyperactivation with adaptive immune suppression.” This pattern aligns with findings from Egozi et al.^9^ and Liu et al.^30^ using different analytical platforms. The macrophage–inflammasome–IL‐1β axis has been recognized as a critical node in NEC, with Shen et al.^31^ demonstrating mTOR/NLRP3/IL‐1β‐mediated inflammatory amplification and Wei et al.^32^ highlighting the protective effects of M1‐to‐M2 polarization. Our gene–immune cell correlations provide molecular annotations for these mechanisms, while single‐cell data showing CCL20 concentrated in macrophages and CXCL5 broadly distributed across cell types indicate that NEC immune dysregulation involves coordinated multi‐cell actions. This suggests that anti‐inflammatory treatment alone may be insufficient, and multi‐target intervention addressing both barrier repair and immune restoration may be more effective.^32^

Virtual knockout analysis provided network‐level evidence for functional differentiation among the three genes. CCL20 and CXCL5 shared extensive downstream perturbation centered on MHC class II molecules and CCR7,^29^ while SLC26A3 knockout produced a distinct profile of epithelial junction and innate immune genes, supporting the “barrier damage‐immune activation” axis. FTH1 was the only gene perturbed across all three knockouts, implicating ferroptosis as a shared convergence point. Geneformer‐based combinatorial perturbation further revealed subadditive rather than synergistic interactions among the three biomarkers, with SLC26A3 as the dominant node, consistent with its upstream barrier‐regulatory role. Extending this analysis to a broader ferroptosis gene panel showed coordinated directional changes across all tested genes, supported by qPCR in the NEC mouse ileum. These findings align with reported GPX4 and SLC7A11 dysregulation in NEC^19^, ^20^ but further indicate that ferroptosis dysregulation operates as a coordinated multi‐node program rather than through isolated single‐gene effects. Combining ferroptosis inhibition with chemokine axis intervention (CCL20‐CCR6 and CXCL5‐CXCR2) may therefore offer a more comprehensive therapeutic approach. However, both virtual knockout and Geneformer perturbation rely on computational inference and require experimental validation.

### 4.1 Study limitations and future perspectives

This study has several limitations. First, the training cohort had a limited sample size, and validation in larger multicenter cohorts is needed. Second, the molecular mechanisms underlying the regulatory relationships among SLC26A3, CCL20, and CXCL5—particularly whether SLC26A3 loss directly regulates CCL20 and CXCL5 transcription through NF‐κB or other shared signaling axes—have not been experimentally dissected. Third, validation was conducted at the mRNA level, and protein‐level detection in clinical samples such as serum or stool has not been assessed. Future research should include prospective clinical evaluation of combined SLC26A3/CCL20/CXCL5 detection for NEC screening, gene knockout models to dissect regulatory networks, and development of bedside rapid detection technologies to advance NEC diagnosis toward clinical application.

## 5 CONCLUSIONS

This study identifies SLC26A3, CCL20, and CXCL5 as core genes closely associated with NEC through integrated WGCNA and machine learning screening, validated in independent cohorts, single‐cell RNA‐seq data, and experimental models. Mechanistically, SLC26A3 downregulation may contribute to disease initiation by disrupting intestinal barrier homeostasis, CCL20 may amplify inflammatory responses through mast cell chemotaxis and antigen presentation regulation, and CXCL5 may exacerbate inflammatory imbalance by recruiting neutrophils while suppressing anti‐inflammatory M2 macrophages. Virtual knockout analysis further revealed that CCL20 and CXCL5 converge on a shared MHC class II network, while SLC26A3 primarily perturbs epithelial barrier and innate immune genes, with ferroptosis emerging as a common convergence point across all three genes. These findings provide candidate molecular markers for early NEC diagnosis and offer a theoretical basis for multi‐target intervention strategies that address both epithelial barrier repair and immune microenvironment rebalancing. Future studies should validate the diagnostic utility of these markers in large‐scale, multicenter clinical cohorts and explore their potential in bedside rapid detection and individualized treatment.

## AUTHOR CONTRIBUTIONS

**Xue Liu**: Conceptualization; methodology; data curation; formal analysis; visualization; writing—original draft. **Wenqiang Sun**: Data curation; formal analysis; methodology; validation. **Zengxue Hu**: Data curation; methodology; validation. **Yihui Li**: Data curation; validation. **Jingtao Bian**: Formal analysis; visualization. **Xueping Zhu**: Conceptualization; supervision; project administration; funding acquisition; writing—review and editing. All authors contributed to the article and approved the submitted version.

## CONFLICT OF INTEREST STATEMENT

The authors declare no conflicts of interest.

## ETHICS STATEMENT

*Human data ethics*: This study involved secondary analysis of publicly available, de‐identified genomic datasets (GSE46619, GSE64801, GSE297483, GSE226086, and PRJNA925809) and a single‐cell RNA‐seq dataset (Egozi et al.^9^; Zenodo: https://doi.org/10.5281/zenodo.5813397) obtained from public repositories. All original studies involving human tissue samples were conducted in accordance with the Declaration of Helsinki and were approved by their respective institutional ethics committees, as described in the original publications. Since our study exclusively utilized publicly available, de‐identified data and did not involve direct contact with human subjects, collection of new biological samples, or access to identifiable personal information, no additional ethical approval was required. *Animal experiment ethics*: All experimental procedures in this study were approved by the Ethics Committee of Soochow University (No. SUDA20241008A01) and strictly adhered to national guidelines and regulations.

## Supporting information

Supporting Information S1

Supporting Information S2

## Data Availability

The original contributions presented in this study are included in the article and Supporting Informations. Further inquiries can be directed to the corresponding author. The publicly available single‐cell RNA‐seq dataset analyzed in this study was obtained from Egozi et al. and is deposited in the Zenodo repository.
