# Supplementary material for: Identification of diagnostic biomarkers for neonatal necrotizing enterocolitis via machine learning screening and single‐cell virtual gene knockout validation
Source: J Cell Commun Signal. 2026 May 28;20(2):e70081. doi: 10.1002/ccs3.70081 (PMC13240151; doi:10.1002/ccs3.70081)
Supplement: Supplementary file 1 — Supporting Information S1 [file CCS3-20-e70081-s002.pdf]

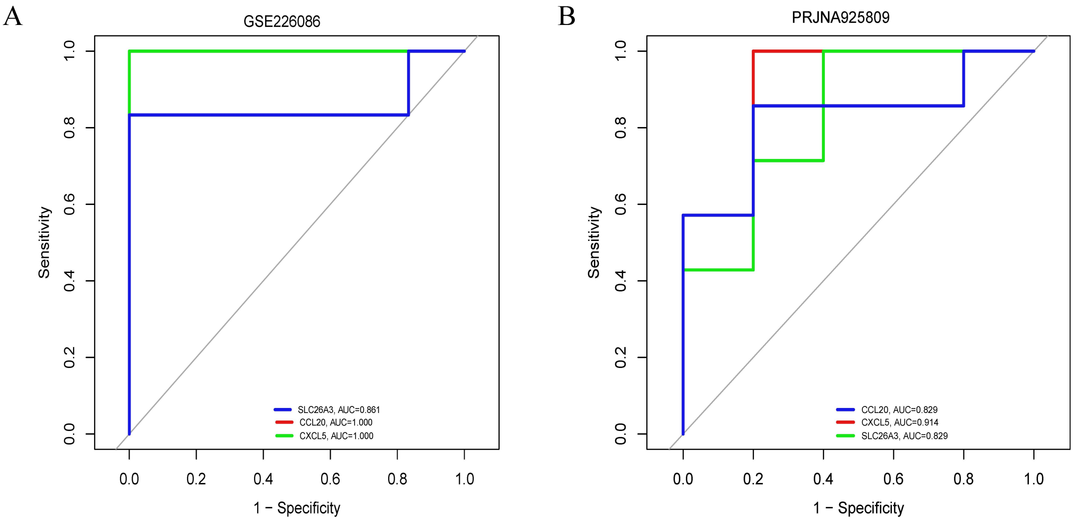

Supplementary Figure S1. ROC Curve Analysis for Validating the Diagnostic Performance of Three Core Biomarkers

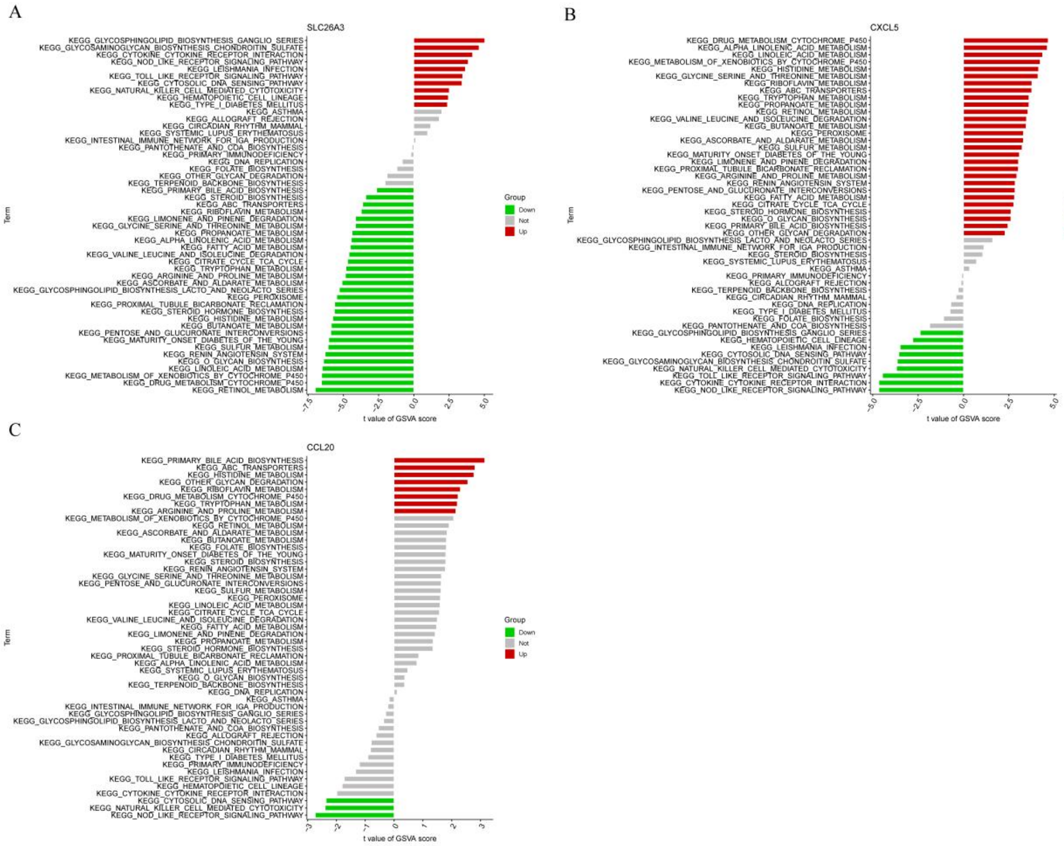

Supplementary Figure S2. GSVA enrichment analysis of core genes.

(A-C) GSVA results indicated that CXCL5, SLC26A3, and CCL20 were enriched in Toll-like receptor signaling, cytokine-cytokine receptor interaction, and NOD-like receptor signaling pathways ($p < 0.05$).

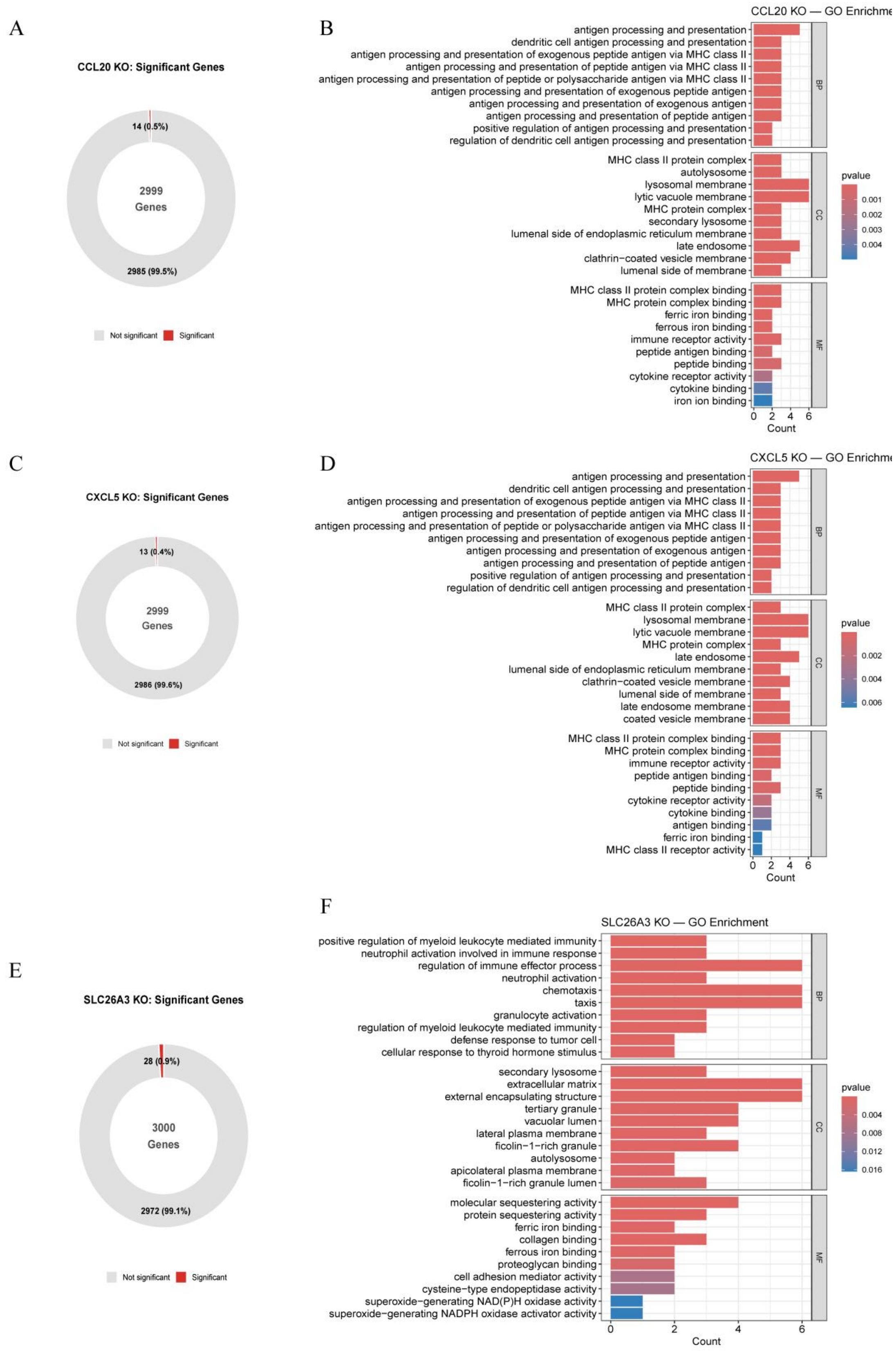

Supplementary Figure S3. Individual virtual knockout results for core genes.
(A–B) CCL20 knockout in NEC: (A) Proportion of significantly perturbed genes (14/2,999, 0.5%); (B) GO enrichment across biological process, cellular component, and molecular function categories. (C–D) CXCL5 knockout in NEC, same layout.

(E–F) SLC26A3 knockout in neonatal controls, same layout.

Supplementary Table 1. The primers used in the present study

Gene	Primer	Sequence (5'-3')
Tnf-α	Forward	GCATGATCCGAGATGTGGAA
	Reverse	TGAGAAGAGGCTGAGGCACA
IL-6	Forward	CCAAGAGGTGAGTGCTTCCC
	Reverse	CTGTTGTTCAGACTCTCTCCCT
Slc26a3	Forward	GGCAAAATGATCGAAGCCATAGGG
	Reverse	GATGGTCCAGGAATGTCTTGTGATGTC
Ccl20	Forward	GCCTCTCGTACATACAGACGC
	Reverse	CCAGTTCTGCTTTGGATCAGC
Cxcl5	Forward	CCGCTGGCATTTCTGTTGCTGT
	Reverse	CAGGGATCACCTCCAAATTAGCG
Fth1	Forward	CAAGTGCGCCAGAACTACCA
	Reverse	ACAGATAGACGTAGGAGGCATAC
Tfrc	Forward	ATGCCGACAATAACATGAAGGC
	Reverse	ACACGCTTACAATAGCCCAGG
GPX4	Forward	AGTACAGGGGTTTCGTGTGC
	Reverse	CATGCAGATCGACTAGCTGAG
SLC7A11	Forward	GGCACCGTCATCGGATCAG
	Reverse	CTCCACAGGCAGACCAGAAAA
ACSL4	Forward	CCTGAGGGGCTTGAAATTCAC
	Reverse	GTTGGTCTACTTGGAGGAACG

Supplementary Table S2. Details of differentially expressed genes in NEC (GSE46619 + GSE64801 + GSE297483). Due to its size (400+ entries), this table is provided as a separate Excel file (Supplementary_Table_S2.xlsx).

Supplementary Table S3. Genes within significant modules identified by WGCNA. Due to its size, this table is provided as a separate Excel file (Supplementary_Table_S3.xlsx).

Supplementary Table S4. Differentially expressed genes within WGCNA modules (intersection of DEGs and WGCNA module genes). Due to its size, this table is provided as a separate Excel file (Supplementary_Table_S4.xlsx).

Supplementary Table S5. GO enrichment analysis of genes at the intersection of WGCNA modules and differential expression. Due to its size, this table is provided as a separate Excel file (Supplementary_Table_S5.xlsx).

Supplementary Table S6. KEGG enrichment analysis of genes at the intersection of WGCNA modules and differential expression. Due to its size, this table is provided as a separate Excel file (Supplementary_Table_S6.xlsx).

Supplementary Table S7. Genes selected by machine learning algorithms. Due to its size, this table is provided as a separate Excel file (Supplementary_Table_S7.xlsx).

Supplementary Table S8. Immune cell correlation analysis. Due to its size, this table is provided as a separate Excel file (Supplementary_Table_S8.xlsx).

Supplementary Table 9. Differential expression of target genes between NEC and neonatal control samples in single-cell RNA-seq analysis

Gene	Mean_NEC	Mean_Neonatal	Log FC	P_value	P_adjusted	Significance	Regulation
SLC26A3	0.0017	0.0043	-0.2985	0.000270917061281302	0.000270917061281302	***	Down in NEC
CCL20	0.2707	0.0675	1.8574	5.05078504760213e-50	7.5761775714032e-50	***	Up in NEC
CXCL5	0.3836	0.0731	2.2437	4.99358403109537e-120	1.49807520932861e-119	***	Up in NEC

Supplementary Table S10. Virtual knockout differentially regulated genes. Due to its size, this table is provided as a separate Excel file (Supplementary_Table_S10.xlsx).

Supplementary Table S11. GO enrichment analysis of virtual knockout perturbed genes. Due to its size, this table is provided as a separate Excel file (Supplementary_Table_S11.xlsx).

Supplementary Table S12. KEGG pathway enrichment of virtual knockout perturbed genes. Due to its size, this table is provided as a separate Excel file (Supplementary_Table_S12.xlsx).

Supplementary Table S13. Mouse Ileum qPCR Raw Data. Due to its size, this table is provided as a separate Excel file (Supplementary_Table_S13.xlsx).

Supplementary Table S14. IEC-6 Cell qPCR Raw Data. Due to its size, this table is provided as a separate Excel file (Supplementary_Table_S14.xlsx).

Supplementary Table S15. Geneformer In Silico Perturbation Results.

Gene	Role	Perturbation	Cell types	Cosine shift toward Neonatal
SLC26A3	Biomarker	Overexpression	Enterocytes	+0.034
FTH1	Anti-ferroptosis	Overexpression	Enterocytes / Macrophages / Fibroblasts	+0.023
SLC7A11	Anti-ferroptosis	Overexpression	Enterocytes / Macrophages / Fibroblasts	+0.020
AIFM2	Anti-ferroptosis	Overexpression	Enterocytes / Macrophages / Fibroblasts	+0.013
GPX4	Anti-ferroptosis	Overexpression	Enterocytes / Macrophages / Fibroblasts	+0.009
TFRC	Pro-ferroptosis	Knockout	Enterocytes / Macrophages / Fibroblasts	+0.007
NCOA4	Pro-ferroptosis	Knockout	Enterocytes / Macrophages / Fibroblasts	+0.006
HMOX1	Anti-ferroptosis	Overexpression	Enterocytes / Macrophages	+0.005

			/ Fibroblasts	
ACSL4	Pro-ferroptosis	Knockout	Enterocytes / Macrophages / Fibroblasts	+0.004
NFE2L2	Anti-ferroptosis	Overexpression	Enterocytes / Macrophages / Fibroblasts	+0.002
CCL20	Biomarker	Knockout	Macrophages	+0.002
CXCL5	Biomarker	Knockout	Enterocytes / Macrophages / Fibroblasts	-0.002

Supplementary Table S16. Mouse Ileum Ferroptosis qPCR Raw Data. Due to its size, this table is provided as a separate Excel file (Supplementary_Table_S16.xlsx).
